# Validation of Multiplex Serology for human hepatitis viruses B and C, human T-lymphotropic virus 1 and *Toxoplasma gondii*

**DOI:** 10.1371/journal.pone.0210407

**Published:** 2019-01-07

**Authors:** Nicole Brenner, Alexander J. Mentzer, Julia Butt, Kathrin L. Braband, Angelika Michel, Katie Jeffery, Paul Klenerman, Barbara Gärtner, Paul Schnitzler, Adrian Hill, Graham Taylor, Maria A. Demontis, Edward Guy, Stephen J. Hadfield, Rachael Almond, Naomi Allen, Michael Pawlita, Tim Waterboer

**Affiliations:** 1 Infections and Cancer Epidemiology, Infection, Inflammation and Cancer Research Program, German Cancer Research Center (DKFZ), Heidelberg, Germany; 2 Faculty of Biosciences, Heidelberg University, Heidelberg, Germany; 3 The Wellcome Centre for Human Genetics, University of Oxford, Oxford, United Kingdom; 4 Big Data Institute, Li Ka Shing Centre for Health Information and Discovery, University of Oxford, Oxford, United Kingdom; 5 Department of Microbiology, Oxford University Hospitals NHS Foundation Trust, Oxford, United Kingdom; 6 NIHR Biomedical Research Centre, Oxford, United Kingdom; 7 Institut für Medizinische Mikrobiologie und Hygiene, Universität des Saarlands, Homburg, Germany; 8 Center for Infectious Diseases, Virology, University Hospital of Heidelberg, Heidelberg, Germany; 9 The Jenner Institute, University of Oxford, Oxford, United Kingdom; 10 Molecular Diagnostic Unit, Imperial College Healthcare NHS Trust, London, United Kingdom; 11 Toxoplasma Reference Unit, Public Health Wales Microbiology, Swansea, United Kingdom; 12 UK Biobank, Stockport, United Kingdom; 13 Nuffield Department of Population Health, Medical Sciences Division, University of Oxford, Oxford, United Kingdom; 14 Molecular Diagnostics of Oncogenic Infections Division, Infection, Inflammation and Cancer Research Program, German Cancer Research Center (DKFZ), Heidelberg, Germany; Centre de Recherche en Cancerologie de Lyon, FRANCE

## Abstract

Multiplex Serology is a high-throughput technology developed to simultaneously measure specific serum antibodies against multiple pathogens in one reaction vessel. Serological assays for hepatitis B (HBV) and C (HCV) viruses, human T-lymphotropic virus 1 (HTLV-1) and the protozoan parasite *Toxoplasma gondii* (*T*. *gondii*) were developed and validated against established reference assays. For each pathogen, between 3 and 5 specific antigens were recombinantly expressed as GST-tag fusion proteins in *Escherichia coli* and tested in Monoplex Serology, i.e. assays restricted to the antigens from one particular pathogen. For each of the four pathogen-specific Monoplex assays, overall seropositivity was defined using two pathogen-specific antigens. In the case of HBV Monoplex Serology, the detection of past natural HBV infection was validated based on two independent reference panels resulting in sensitivities of 92.3% and 93.0%, and specificities of 100% in both panels. Validation of HCV and HTLV-1 Monoplex Serology resulted in sensitivities of 98.0% and 95.0%, and specificities of 96.2% and 100.0%, respectively. The Monoplex Serology assay for *T*. *gondii* was validated with a sensitivity of 91.2% and specificity of 92.0%. The developed Monoplex Serology assays largely retained their characteristics when they were included in a multiplex panel (i.e. Multiplex Serology), containing additional antigens from a broad range of other pathogens. Thus HBV, HCV, HTLV-1 and *T*. *gondii* Monoplex Serology assays can efficiently be incorporated into Multiplex Serology panels tailored for application in seroepidemiological studies.

## Introduction

Multiplex Serology is a high-throughput methodology allowing for the simultaneous detection of serum antibodies against multiple pathogens in one reaction vessel by performing different pathogen-specific assays in parallel. This is achieved by bead-based presentation of selected antigens to primary serum antibodies [1]. Formation of immunocomplexes consisting of the respective antigen, bound primary antibodies and anti-human detection antibodies can be quantitatively measured using a Luminex flow cytometer distinguishing up to 100 bead sets (i.e., antigens). The term ‘Monoplex Serology’ is used to describe pathogen-specific serological assays including only antigens of the respective pathogen, while Multiplex Serology is defined as the combination of at least two Monoplex Serology assays. We have successfully developed Monoplex Serology assays for human papillomaviruses, *Helicobacter pylori*, human polyomaviruses, and human herpesviruses in order to perform seroepidemiological studies [1, 2, 3, 4]. To expand our portfolio of serological assays for infectious agents known or suspected to be involved in cancer and / or neurodegenerative disease development, we developed and validated pathogen-specific Monoplex Serology assays for hepatitis B (HBV) and C (HCV) viruses, human T-cell lymphotropic virus 1 (HTLV-1) and the protozoan parasite *Toxoplasma gondii* (*T*. *gondii*) for application in Multiplex Serology.

HBV is a hepatotropic DNA virus belonging to the family of *Hepadnaviridae*, while HCV is a hepatotropic RNA virus and is classified as a member of the *Flaviviridae* family [5]. Transmission of HBV and HCV mainly occurs via contaminated blood products, vertical transmission, injection drug use or sexual intercourse [6, 7]. Infection by HBV or HCV may be asymptomatic or symptomatic. Acute HBV infection is usually cleared by the adult host but may lead to chronic infection in approximately 5% of cases, whereas 90% of those infected in the neonatal period develop a chronic infection [8, 9]. Approximately 60–90% of HCV infected individuals develop a chronic HCV infection [10, 11, 12]. Both HBV and HCV can cause chronic liver disease in persistently infected individuals, some of whom may develop liver cirrhosis and / or hepatocellular carcinoma (HCC). Thus, HBV and HCV have been classified as group I human carcinogens for HCC [13, 14]. In addition, both viruses have been associated with non-Hodgkin lymphoma and cholangiocarcinoma [13, 14]. The prevalence of chronic HBV and HCV infection and their attributable fractions in liver cancer vary by region and human development index [15, 16]. The worldwide prevalence of chronic HBV infection is 3.6%, with estimates ranging from less than 0.5% in some European and American countries like Norway, France, the UK or US, to more than 20% in South Sudan [15]. Global viraemic HCV prevalence was estimated to be 1.1% ranging between 0.4% in some European countries such as Germany, Denmark or the UK, to 10% in Egypt and Gabon [17]. In 2012, the global attributable fractions of liver cancer were 56% and 20% for HBV and HCV, respectively [16]. Chronic HBV or HCV infection can be treated with different antiviral drugs, which in case of HCV can now produce long-term viral eradication in most cases suggesting a reduction in mortality [18, 19]. Eradication of chronic HBV is not achievable at present [20]. Prophylactic vaccination is available for HBV but not for HCV [13, 21, 22]. Infection history for both viruses can be detected via serology [7, 23]. In the case of HCV, additional tests to detect HCV RNA or antigen are needed to accurately distinguish between current and past infection [7].

HTLV-1 belongs to the group of delta-type retroviruses. Transmission may occur via contaminated blood, sexual intercourse, injection drug use and vertical transmission [24]. This retrovirus has been classified as a group I carcinogen for adult T-cell leukaemia/lymphoma in humans [13, 14]. Additionally, it is known to cause HTLV-1-associated myelopathy/tropical spastic paraparesis [13, 24, 25]. Global HTLV-1 prevalence is generally low (<1%), except for endemic areas, such as certain regions in southwestern Japan, sub-Saharan Africa, South America and the Caribbean islands where a HTLV-1 prevalence of up to 20% was reported [13, 24]. Currently, neither a vaccine against HTLV-1 infection nor any treatment for virus clearance is available. Treatment focuses on HTLV-1 induced diseases, which only occur in a small fraction of infected individuals (≤5%) [13, 26, 27, 28]. HTLV-1 infection is currently diagnosed by a combination of a serological assay and a confirmation assay such as PCR or immunoblotting [24].

*T*. *gondii* is an obligate intracellular protozoan parasite infecting warm-blooded animals including humans as intermediate hosts. The parasite’s definitive hosts are felines. Multiple routes of human infection have been reported, including ingestion of contaminated water or undercooked meat, blood products, transplants and congenital infection [29, 30]. Upon infection, *T*. *gondii* causes toxoplasmosis that may result in flu-like symptoms in immunocompetent individuals or severe disease in immunocompromised individuals, neonates and congenitally infected foetuses [29, 31]. In addition, infection by *T*. *gondii* has been associated with impacts on human behaviour and disorders such as schizophrenia [32, 33]. The necessity for treatment depends on the severity of toxoplasmosis and the immune status of the infected individual [34, 35]. No prophylactic or therapeutic vaccine against *T*. *gondii* is available. It has been estimated that the global *T*. *gondii* prevalence is approximately 30% [29, 36]. Serological methods are often used for diagnosis of toxoplasmosis. In addition, molecular methods such as PCR have been developed to detect DNA in different biospecimens [31].

The above mentioned pathogens have one common denominator–infected individuals can be detected by serology. In case of HBV, serology allows assessing an individual’s infection and vaccination history. Serological assays can be deployed in seroepidemiological studies to investigate associations between infections and diseases, such as certain types of cancer or neurodegenerative diseases. Thus, we developed pathogen-specific Monoplex Serology assays to detect natural HBV, HCV, HTLV-1 and *T*. *gondii* infections. As a secondary aim, HBV Monoplex Serology was aimed to additionally investigate HBV vaccination history.

The developed assays were validated against reference assays used for clinical diagnosis. Selection of a minimum number of pathogen-specific antigens reaching optimum statistical characteristics in comparison to the respective reference assays was pursued for efficient application in Multiplex Serology. Here, we report the successful validation of Monoplex Serology assays against HBV, HCV, HTLV-1 and *T*. *gondii* comprising as little as two antigens each and their incorporation into Multiplex Serology.

## Materials and methods

### Antigen selection and cloning for recombinant antigens expressed in *Escherichia coli*

In Table 1, antigens selected for development and validation of pathogen-specific Monoplex Serology assays are shown. Proteins and their amino acid sequences were chosen based on reported immunogenicity in the literature. Recombinant proteins were expressed in *Escherichia coli* (*E*. *coli*) without reported signal peptides or transmembrane regions to facilitate efficient bacterial expression. The sequences for *T*. *gondii* protein bag-1 and the hepatitis B surface antigen (HBs) were codon-optimized for expression in *E*. *coli* and assembled into a modified pGEX4T3 vector (pGEX4T3tag, described below) by gene synthesis (eurofins Genomics, Ebersberg, Germany) [37]. Sequences for the cloned antigens were obtained from DNA vectors or extracted from parasite cells (Table 1). Antigen expression for HCV was previously described [38]. For cloning of antigen sequences into pGEX4T3tag vectors, corresponding primers were designed and ordered from eurofins Genomics. The antigen constructs were then amplified in *E*. *coli* DH5α. The inserts were sequenced (eurofins Genomics / GATC Biotech, Konstanz, Germany) and aligned with reference sequences (NCBI nucleotide database) for validation of correct sequences (amino acid changes listed in Table 1).

**Table 1:** Characteristics of selected pathogen-specific serology antigens.

pathogen-specific antigen	function	GST-X-tag fusion protein*	aa	*E*. *coli* strain	codon optimized^1^	DNA template / accession no. Uniprot	NCBI reference (nucleotide range)
**HBV**							
HBc	core antigen	yes	1–185	B	-	HBV 1.28x genome (genotype A, subtype adw2)^2^ [39]	AM282986.1 (1901-2455 bp)
HBe	soluble nucleocapsid associated antigen	yes	20–179^3^	B	-		KY003230.1 (1871–2347 bp)
HBs	surface antigen	yes	99–169^4^ [40]	B	X	Q17UT6	-
HBs from human plasma^5^	surface antigen	no	-	-	-	-	-
recombinant HBs from *S*. *cerevisiae*^6^	surface antigen	no	-	-	-	-	-
HBs VLP^7^	surface antigen	no	-	-	-	-	-
**HCV (subtype 1a)**							
Core	structural antigen	yes	full length^8^	R	-	pCV-H77^8^	-
NS3	protease and RNA helicase activity	yes		R	-		-
NS4A	cofactor for NS3	yes		B	-		-
NS5A	phosphoprotein, virus assembly	yes		B	-		-
NS5B	RNA-dependent RNA polymerase	yes		R	-		-
**HTLV-1**							
gag	structural antigen	yes	1–429	R	-	isolate MT-2^10^	X15951.1
env	structural antigen	yes	21–312^9^	B	-		X56949.2
tax	regulatory protein	yes	1–353	B	-		AAF71373.1^11^
rex	regulatory protein	yes	1–372	B	-		AF033817.1^11^** (4773–4833 bp, 6950–8008 bp)
HBZ	regulatory protein	yes	1–206	R	-		BAX34773.1^11^
***T*. *gondii***							
sag1	surface antigen	yes	31–349^3^	B	-	DNA extracted from parasites (strain ME49)^12^	XP_002368205.2
p22	surface antigen	yes	27–187^3^	R	-		XM_018781602.1
bag1	bradyzoite specific protein	yes	exons 1–4 (229 aa)	B	X	Q27354**	-

^1^*T*. *gondii* bag-1 and HBV HBs expression plasmids were obtained via gene synthesis and sequence identity confirmed by manufacturer.

^2^ kindly provided by C-T. Bock, Robert Koch Institute, Berlin, cloned from pHBV-1.2 containing synthetic HBV 1.28x genome (AF305422; HBV A adw2).

^3^ transmembrane domain / signal peptide excluded

^4^ sequence was reported to be the major hydrophilic region and to harbour B-cell epitopes

^5^ HBs from human plasma, HBsAG (ad) protein (Fitzgerald Industries International, Acton, USA; Cat.No: 30-AH16U 0.9 mg/ml, >95% pure)

^6^ HBs expressed from *Saccharomyces cerevisiae* (BIO TREND, Destin, USA)

^7^ kindly provided by A. Hill (The Wellcome Centre for Human Genetics, University of Oxford, Oxford, UK; The Jenner Institute, University of Oxford, Oxford, UK)

^8^ details on antigens were previously published by Dondog *et al*. [38]

^9^ exclusion of leader peptide and gp21 (starts after cleavage site at aa 312–313)

^10^ obtained from Biotech Research Laboratories, Rockville, Maryland, USA, provirus genome from T-cell leukemia cell line MT-2

^11^ no NCBI reference sequence for HTLV-1 strain MT-2 available

^12^ kindly provided by U. Groß and W. Bohne, Institute for Medical Microbiology, University of Göttingen, Germany

* Antigens were recombinantly expressed in E.coli as GST-X-tag fusion proteins with an N-terminal GST and a C-terminal SV40 tag (last 11 aa of large T-antigen).

** Upon alignment with the NCBI / Uniprot amino acid sequences the following deviations were observed. HTLV-1 rex: K212E, A240V, L362P; *T*. *gondii* bag-1: P3L

aa: amino acids

B: *E*. *coli* strain BL21

R: *E*. *coli* strain BL21 Rosetta

### Antigen expression in *E*. *coli*

Non-commercial recombinant antigens were expressed in either *E*. *coli* strain BL21 or BL21 Rosetta (Table 1). Antigens were expressed as GST-X-tag fusion proteins with an N-terminal GST and a C-terminal SV40-tag consisting of the last 11 amino acids of the large T-antigen (tag) [37]. The expression vector pGEX4T3tag contains an ampicillin resistance gene (amp^R^) enabling positive selection of transformed colonies. Transcription is inducible by IPTG. Bacterial cells were lysed and cleared. Glycerol (50% (v/v)) was added to the lysates, and they were stored at -20°C. Lysates underwent quality control as described before including Coomassie staining, western blot staining for N- and C-terminal tags and GST capture ELISA as described elsewhere [37, 38].

### Covalent coupling of HBs antigens to polysterene beads

Three different HBs products were obtained from external providers (Table 1). These lack an N-terminal GST-tag and were thus covalently coupled to polystyrene beads as described elsewhere [1]. The commercial HBs purified from serum, or recombinantly expressed in *S*. *cerevisiae* were coupled in a concentration of 100 μg/ml. Virus-like particles (VLPs, purified into phosphate buffered saline; Indian Immunologicals LTD., Hyderabad, Telangana, India) were coupled in a concentration of 50 μg/ml to the beads. Prior to coupling, confirmation of VLP structure was undertaken using dynamic light scattering (Zetasizer nano ZS, Malvern Panalyical Ltd., Malvern, UK). The average measured polydispersity index of the six duplicate samples of HBs antigen (HBsAg) was around 0.3 which suggested a degree of polydispersity (using a polydispersity index of less than 0.3 suggesting monodispersed samples). Volume size distribution demonstrated that the majority (>98%) of particles were in the size range of 30 nm confirming preserved VLP structure.

### Reference panels and reference assays

Pathogen-specific Monoplex Serology assays were validated against commercial reference assays based on reference panels (RP Ia,b; II-IV; Va,b,c) listed in Table 2. Sera for RP Ia and Va were obtained from the routine diagnostic laboratory of the Saarland University Medical School. The sera were stored at -20°C. Both serum panels are overlapping and comprise n = 230 sera in total of which five sera were not tested for anti-HBs antibodies at the German Cancer Research Center (DKFZ, Heidelberg). Of the 139 anti-HBc negative sera of panel Ia, 110 were tested anti-HBs negative and 29 were tested anti-HBs positive by the Saarland University Medical School. Of the 91 anti-HBc positive sera, 29 were tested negative for anti-HBs and 62 tested positive for anti-HBs by the reference laboratory.

**Table 2:** Characteristics of reference serum panels.

pathogen	provider	panel	n Ref+	n Ref-	reference assay
HBV	B. Gärtner (Institut für Mikrobiologie und Hygiene, Saarland University Hospital, Homburg, Germany)	Ia	91	139	ARCHITECT anti-HBc (Abbott Laboratories, Abbott Park, USA)
HBV	K. Jeffery (Oxford University Hospitals NHS Trust, Department of Microbiology)	Ib	57	100	ARCHITECT anti-HBc II (Abbott Laboratories)
HCV	K. Jeffery	II	50*	104**	ARCHITECT Anti-HCV (Abbott Laboratories), confirmatory ELISA Murex anti-HCV (Diasorin, Saluggia, Italy)*
HTLV-1	G. Taylor (Imperial College Healthcare NHS Trust, Molecular Diagnostic Unit)	III	100	100	ARCHITECT rHTLV-I/II (Abbott Laboratories), confirmation and typing by Genelabs Diagnositics HTLV 2.4 assay (Genelabs, Redwood City, USA)
*T*. *gondii*	E. Guy (Public Health Wales Microbiology, Toxoplasma Reference Unit)	IV	148	50	Sabin-Feldman dye test
HBV anti-HBs	B. Gärtner	Va***	90	135	ARCHITECT anti-HBs (Abbott Laboratories)
	P. Schnitzler (Center for Infectious Diseases, Virology, University Hospital of Heidelberg, Heidelberg, Germany)	Vb	55	54	ARCHITECT AUSAB (Abbott Laboratories)
		Vc	72	64	ADVIA Centaur Anti-HBs2 (Siemens Healthcare Diagnostics Inc., Tarrytown, NY, USA)

n Ref+: number of reference assay positives

n Ref-: number of reference assay negatives

* Determined by double seropositivity to the ARCHITECT Anti-HCV and the Murex anti-HCV assay.

** Including 4 sera classified as false-positive by the confirmatory assay (single positive to ARCHITECT Anti-HCV assay). These sera were classified as HCV reference serostatus negative in statistical analysis.

*** Serum panel overlaps with reference panel Ia.

Anonymised sera for RP Ib and II were obtained from the routine diagnostic laboratory of Oxford University Hospitals; each sample had been tested for HBV or HCV, respectively. These sera were stored at -20°C and were reaching the end of their retention period. RP Ib comprised samples tested negative to anti-HBc (n = 100) that were also tested negative for the presence of HBsAg. Of the 57 anti-HBc positive reference samples, 54 were tested negative for HBsAg and 3 tested positive for HBsAg. HCV positive sera of RP II (n = 50) were tested double positive by the ARCHITECT Anti-HCV assay and the Murex anti-HCV assay. These sera were additionally tested for HCV antigen (ARCHITECT HCV Ag, Abbott) of which 22 tested HCV antigen negative and 28 HCV antigen positive. Four sera tested positive by the ARCHITECT Anti-HCV assay are considered false-positive, as the confirmatory assay (Murex anti-HCV assay) was negative.

RP III consisted of sera donated by patients who attended the National Centre for Human Retrovirology to the Communicable Diseases Research Tissue Bank (approved by the National Research Ethics Service: Ref 15/SC/0089). Processing and storage was conducted by the department of Retrovirology and GU Medicine, Division of Infectious Diseases, Imperial College London.

The sera of RP IV were selected from a prospective serum cohort tested with the Sabin-Feldman Dye Test. Negative or positive serostatus was determined with the dye test (cut-off level of 2 IU/ml), with the assay calibrated against the WHO ToxoG International Standard Preparation [41]. The sera were stored at -20°C.

RP Vb and Vc were received from the routine diagnostic testing of the Center for Infectious Diseases at the University Hospital of Heidelberg. The reference sera were tested at the University Hospital of Heidelberg in 2014 (Vb) and 2016 (Vc) and stored at -20°C until testing at the DKFZ.

### Monoplex and Multiplex Serology

Serum antibodies against selected antigens (Table 1) were measured using pathogen-specific Monoplex Serology and Multiplex Serology as previously reported [1]. In brief, antigens were expressed as GST-X-tag fusion proteins and *in situ* affinity purified from crude lysates on glutathione casein-coated fluorescence-labelled polystyrene beads (carboxylated xMAP Microspheres, Luminex Corp. Austin, Texas, USA) except for externally obtained HBs antigens. These were covalently coupled to fluorescence-labelled polystyrene beads as described above. The polystyrene beads contain two fluorescent dyes in different ratios. Thus, up to 100 different bead sets can be distinguished by the Luminex flow cytometer. Antibodies against multiple antigens were measured in one reaction vessel by loading each antigen onto another bead set and subsequent combination into a single bead mix. In Monoplex Serology, only the pathogen-specific antigens described in Table 1 plus antigen GST for background correction were included in the bead mix. In Multiplex Serology, antigens from multiple pathogen-specific Monoplex Serology assays plus antigen GST were combined into the bead mix. Serum was incubated with the antigen-loaded beads and primary antibodies were detected using a biotinylated goat-α-human IgM/IgG/IgA secondary antibody (1:1000, #109-065-064, Jackson Immunoresearch, West Grove, PA, USA). Subsequent incubation with a reporter dye, streptavidin-R-phycoerythrin (1:750, PE-Streptavidin Conjugate, MOSS Inc., Pasadena, CA, USA), allowed quantitative measurement of the formed immunocomplexes in a Luminex 200 flow cytometer. Measuring at least 100 beads per bead set, Median Fluorescence Intensities (MFI) were calculated. Pathogen-specific Monoplex Serology was performed in dilutions 1:100 and 1:1000 on each reference panel. For HBV, HCV and HTLV-1, results for dilution 1:1000 are reported, while for *T*. *gondii* the optimum dilution 1:100 is reported.

Multiplex Serology incorporating HBV, HCV, HTLV-1 and *T*. *gondii* Monoplex Serology assays was performed at a 1:1000 serum dilution including additional pathogen-specific assays (e.g. human herpesviruses 1–8, human polyomaviruses, human papillomaviruses, human immunodeficiency virus 1).

### Statistical analyses

Sera were received and tested in a blinded fashion. After testing, the Wellcome Centre for Human Genetics served as trusted third party, and, following confirmation that preliminary sensitivity and specificity metrics were greater than 85%, merged testing results obtained at DKFZ with the previously provided reference data, thus unblinding the analysis. Antibody measurements were then assessed by reference serostatus and refined cut-offs were determined in a stepwise approach. Analogous to Receiver Operation Characteristics analysis, sensitivity and specificity were optimized to achieve at least 85% by gradually increasing the cut-off starting from a minimum of 30 MFI at serum dilution 1:1000, and 50 MFI at 1:100. Final cut-offs were determined favouring specificity until an additional increase in specificity resulted in a disproportionate loss in sensitivity, thus maximizing agreement as a secondary criterion.

In addition to sensitivity and specificity, Cohen’s *kappa* (*k*) plus corresponding 95% confidence intervals (CI) were calculated using SAS 9.4. The overall serostatus for each pathogen based on multiple pathogen-specific antigens was evaluated by systematic combination (e.g. AND/OR algorithms) of the antigens optimizing the statistical characteristics using SAS 9.4. *Kappa* statistics were evaluated as follows: 0.01≤*k*≤0.20: slight agreement, 0.21≤*k*≤0.40: fair agreement, 0.41≤*k*≤0.60: moderate agreement, 0.61≤*k*≤0.80 substantial agreement, and 0.81≤*k*≤0.99: almost perfect agreement [42]. Statistical performance of Multiplex Serology compared to the corresponding reference panel was calculated analogous to the procedure described for Monoplex Serology.

Comparison of Monoplex and Multiplex Serology performance for the corresponding reference panels was conducted by calculating Intraclass Correlation Coefficients (ICCs) using R 3.5.0, package ‘psych’ [43]. ICC(3,1) plus corresponding 95% CI. ICCs were evaluated as follows: 0.01≤ICC≤0.49: poor reliability, 0.50≤ICC≤0.74: moderate reliability, 0.75≤ICC≤0.89: good reliability, 0.90≤ICC≤1.00: excellent reliability [44]. The numbers of sera slightly differed for statistical comparisons from the numbers given in Table 2 by 0–4 sera for RP Ia/b-III and by 27 sera for RP IV due to insufficient bead count during measurements or insufficient sample volume.

## Results

### Antigen selection

The HBV, HCV, HTLV-1 and *T*. *gondii* pathogen-specific Monoplex Serology assays initially comprised 3–5 preselected antigens. For all pathogens, selection took place according to reported antigen immunogenicity (e.g. [38, 45, 46, 47, 48, 49, 50, 51, 52]) and commercial assays. For HBV, infection and/or vaccination status and the associated serological antibody patterns were additionally taken into account. Characteristics of the selected antigens are shown in Table 1. All non-commercial recombinant antigens were expressed as GST-fusion proteins as described previously [37]. Parental plasmids used for antigen expression were sequenced to confirm correct amino acid sequences. Most antigens did not show any variation from the reference sequence. Amino acid changes resulting from nucleotide mismatches are reported in Table 1.Three amino acid changes were found for HTLV-1 rex in comparison with the reported NCBI reference and one for *T*. *gondii* bag1 (Table 1).

### Comparison of pathogen-specific Monoplex Serology assays with reference serostatus

**Fig 1:**
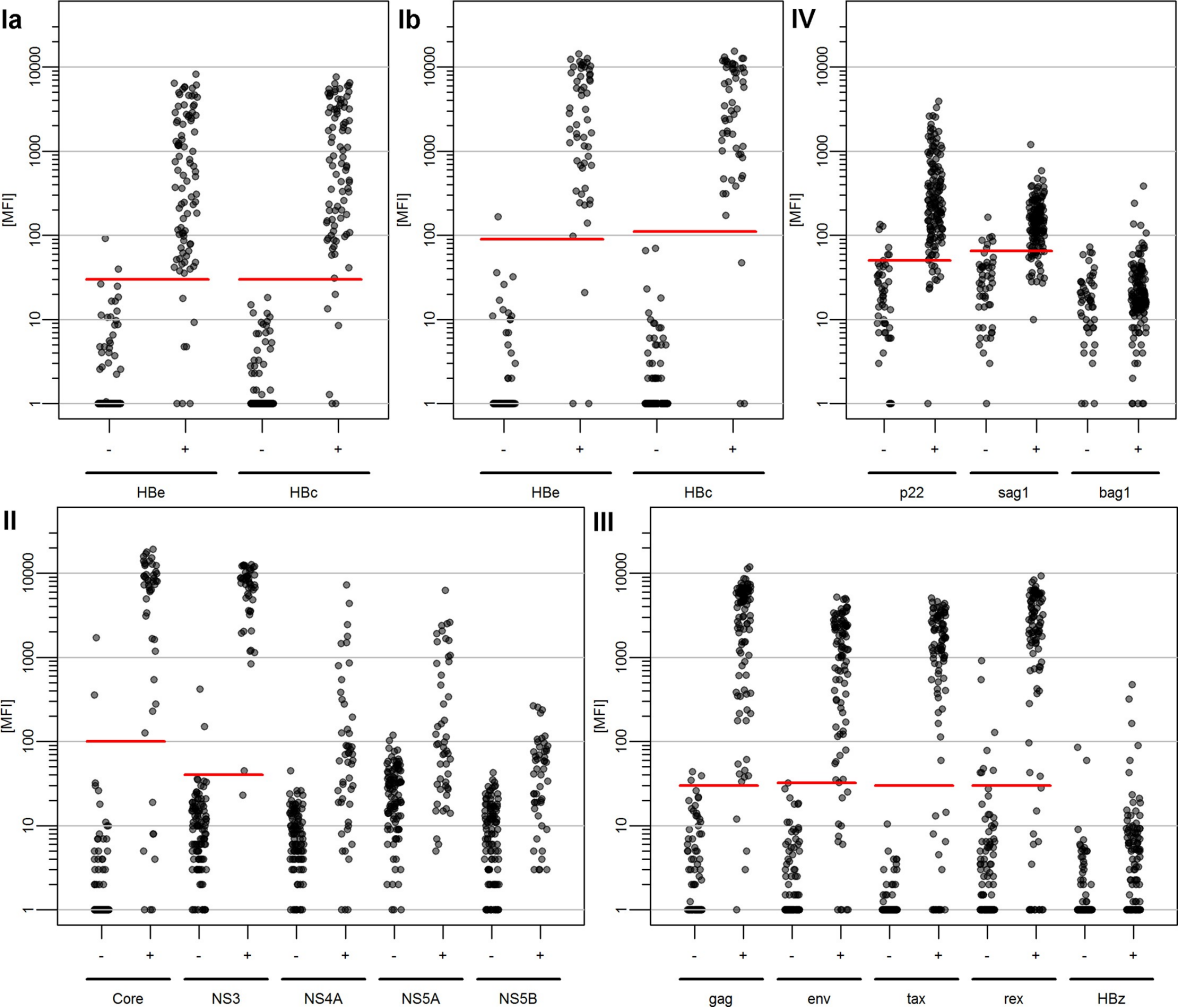
Comparison of quantitative antibody measurements (MFI) with reference serostatus in pathogen-specific Monoplex Serology for HBV (Ia, Ib), HCV (II), HTLV-1 (III) and *T*. *gondii* (IV). Ia, Ib, II-IV indicate corresponding reference panels. red lines: optimized cut-offs for single antigen performance; cut-offs were determined by optimizing specificity and sensitivity analogous to Receiver Operator Characteristics analysis. No cut-offs were determined for antigens that showed a reduced capacity to distinguish between reference assay positives and negatives. MFI: Median Fluorescence Intensities.

Five reference serum panels (denoted RP Ia, Ib and II-IV) were used for the evaluation of the Monoplex Serology assays. Measured antibody reactivities (MFI) stratified by reference assay status are shown in Fig 1. In each Monoplex Serology assay, at least three different antigens were included. Cut-offs for single antigen performance were determined by optimizing specificity and sensitivity. Statistical characteristics of the single antigens are shown in Table 3. Overall pathogen-specific seropositivity was evaluated by systematic combination of all included antigens for each pathogen-specific assay. Cut-offs for overall pathogen-specific performance were determined identically as for single antigens, and statistical characteristics for overall seropositivity in Monoplex Serology are shown in Table 4.

**Table 3:** Single antigen performance in Monoplex Serology in corresponding reference panels.

RP	antigen	cut -off^1^ (MFI)	specificity (95% CI)	sensitivity (95% CI)	*kappa* (95% CI)
Ia	HBV				
	HBe	30	98.6 (94.9–99.8)	92.3 (84.8–96.9)	0.92 (0.86–0.97)
	HBc	30	100.0 (97.4–100.0)	93.4 (86.2–97.5)	0.94 (0.90–0.99)
Ib	HBV				
	HBe	90	99.0 (99.5–100.0)	93.0 (83.0–98.1)	0.93 (0.87–0.99)
	HBc	110	100.0 (96.4–100.0)	93.0 (83.0–98.1)	0.94 (0.89–1.00)
II	HCV				
	Core	100	98.1 (93.2–99.8)	84.0 (70.9–92.8)	0.85 (0.76–0.94)
	NS3	40	98.1 (93.2–99.8)	98.0 (89.4–100.0)	0.96 (0.91–1.00)
III	HTLV-1				
	gag	30	97.0 (91.5–99.4)	96.0 (90.1–98.9)	0.93 (0.88–0.98)
	env	32	100.0 (96.4–100.0)	88.0 (80.0–93.6)	0.88 (0.81–0.95)
	tax	30	100.0 (96.4–100.0)	81.0 (71.9–88.2)	0.81 (0.73–0.89)
	rex	30	92.0 (84.8–96.5)	83.0 (74.2–89.8)	0.75 (0.66–0.84)
IV	T. gondii				
	p22	50	86.0 (73.3–94.2)	92.6 (87.1–96.2)	0.77 (0.66–0.87)
	sag1	65	86.0 (73.3–94.2)	84.5 (77.6–89.9)	0.64 (0.52–0.75)

^1^ cut-offs determined optimizing specificity and sensitivity analogous to Receiver Operator Characteristics analysis

CI: confidence interval

RP: Reference panel

MFI: median fluorescence intensity

**Table 4:** Overall pathogen-specific statistical performance in Monoplex Serology in corresponding reference panels.

RP	pathogen	antigen	cut -off^1^ (MFI)	algorithm	specificity (95% CI)	sensitivity (95% CI)	*kappa* (95% CI)
Ia	HBV	HBe	30	AND	100.0 (97.4–100.0)	92.3 (84.8–96.9)	0.94 (0.89–0.98)
		HBc	30				
Ib	HBV	HBe	90	AND	100.0 (96.4–100.0)	93.0 (83.0–98.1)	0.94 (0.89–100.0)
		HBc	110				
II	HCV	Core	100	AND / OR	96.2 (90.4–98.9)	98.0 (89.4–100.0)	0.93 (0.86–0.99)
		NS3	40				
		Core	30	AND	100.0 (96.5–100.0)	84.0 (70.9–92.8)	0.88 (0.79–0.96)
		NS3	30				
III	HTLV-1	gag	50	AND / OR	100 (96.4–100.0)	94.0 (87.4–97.8)	0.94 (0.89–0.99)
		env	50				
IV	*T*. *gondii*	p22	77	AND / OR	92.0 (80.8–97.8)	91.2 (85.5–95.2)	0.79 (0.69–0.88)
		sag1	100				

^1^ cut-offs determined by optimizing specificity and sensitivity

CI: confidence interval

RP: Reference panel

MFI: median fluorescence intensity

#### HBV Monoplex Serology validation

HBV Monoplex Serology for the detection of natural infection history based on antigens HBc and HBe was validated using two independent reference panels (Ia and Ib). Stratification of measured antibody reactivities by reference assay status revealed high concordance for HBc and HBe with both reference assays (Fig 1 Ia/Ib). For RP Ia, two false-positives (1%) for HBe and six (7%) and seven (8%) false-negatives for HBc and HBe, respectively, were observed. Similarly, only one false-positive (1%) for HBe and three false-negatives (5%) each for HBe and HBc were detected for RP Ib. Statistical characteristics for the single antigens reveal successful assay validation with both reference panels (Table 3). Sensitivities ranged between 92.3% and 93.4%, and specificities between 98.6% and 100.0%. *Kappa* statistics ranged between 0.92 and 0.94 showing almost perfect agreement. Overall HBV seropositivity was best determined by a combination of both HBc and HBe, or HBc alone. For both reference panels, the combination of HBc and HBe resulted in 100.0% specificity and a sensitivity of 92.3% in case of RP Ia and 93.0% on RP Ib (Table 4). Similar statistical characteristics were observed by an AND/OR combination of HBc and HBe (not shown) consistent with almost perfect correlation between measured HBc and HBe antibody reactivities (r_RPIa_ = 0.98, r_RPIb_ = 0.99, S1 Fig Ia/Ib).

In addition, anti-HBs serology was pursued to be established and validated based on RP Va. However, multiple attempts to establish and to successfully validate anti-HBs measurements in Monoplex Serology failed on our platform (S2 Fig). These attempts included using a GST-HBs-tag fusion protein expressed in *E*. *coli*, purified HBs antigen from human plasma (not shown due to high background reactivity), recombinant HBs antigen from *Saccharomyces cerevisiae*, and HBV virus-like particles (VLP) also used in vaccination programmes to protect against HBV (S2A–S2C Fig). Due to insufficient sensitivity and/or high background reactivity in reference assay negatives, no cut-offs were applied and no statistical characteristics calculated. In addition to RP Va, two additional reference panels (Vb, Vc) were used to evaluate the performance of the covalently coupled HBV VLPs. However, this experiment confirmed the poor capacity to distinguish between anti-HBs reference positives and negatives already observed on RP Va (S2D Fig).

#### HCV Monoplex Serology validation

HCV Monoplex Serology was previously validated by Dondog *et al*. against a meanwhile outdated reference assay [38]. Here, we report a contemporary validation based on a different reference panel (RP II) using the ARCHITECT Anti-HCV assay in combination with a confirmatory ELISA (Murex anti-HCV, Diasorin) used to detect false-positives. HCV Monoplex Serology is based on the Core protein and non-structural (NS) proteins NS3, NS4A, NS5A and NS5B expressed from HCV subtype 1a. HCV antigens Core and NS3 showed high capacity to discriminate between reference assay negatives and positives (Fig 1II). However, antigen Core was less sensitive compared to antigen NS3, detecting eight (16%) compared to one (2%) false-negatives, respectively. Both antigens detected only two false-positives (2%). Thus, both Core and NS3 showed high specificities of 98.1% and sensitivities of 98.0% in the case of NS3 and 84.0% for Core (Table 3). This resulted in almost perfect agreement with the reference assay for both antigens (*k*_*NS3*_ = 0.96, *k*_*Core*_ = 0.85). NS4A, NS5A and NS5B were less capable of distinguishing between reference assay positives and negatives and no cut-offs were applied. Only a subset of reference assay positive sera showed antibody reactivity against these antigens. However, detection of antibodies by the additional non-structural proteins was highly specific as almost all reactivities in reference assay negative sera were <100 MFI while reference assay positive sera yielded up to 10,000 MFI (Fig 1II).

Different criteria for overall HCV seropositivity based on multiple HCV antigens were applied (Table 4). Optimum statistics were reached by an AND/OR combination of Core and NS3 yielding a specificity of 96.2% and a sensitivity of 98.0%. In Dondog *et al*., an AND combination of Core and NS3, or a combination of NS3 with two other non-structural proteins were identified as optimal for overall HCV seropositivity [38]. These criteria were also applied but shown to be less sensitive for analysis of RP II (Table 4, S3 Fig, combinations with other non-structural proteins not shown).

### HTLV-1 Monoplex Serology validation

The initial HTLV-1 Monoplex Serology panel included antigens gag, env, tax, rex and HBz and was validated against a combination of two reference assays used as standard procedure in the UK to diagnose HTLV-1 infection on RP III (Table 2). All antigens except HBz distinguished reference assay positive from negative serum samples (Fig 1 III). For gag, env and tax, zero to three (0–3%) false-positives were detected. The rex antigen detected a higher number of false-positives (n = 8; 8%). The number of false-negatives ranged between four for gag (4%) and 19 for tax (19%). Of the 100 reference assay positives, 76 were seropositive to all four informative HTLV-1 antigens (gag, env, tax, rex), six sera to three out of four antigens, and nine sera to gag and env. Only two reference assay positive sera (2%) did not react with any of the included HTLV-1 antigens. The resulting specificity was high for all four antigens ranging between 92.0% for rex and 100.0% for env and tax (Table 3). Sensitivity was lowest for tax (81.0%) and highest for gag (96.0%). *Kappa* statistics varied from substantial agreement for antigen rex (*k* = 0.75) to almost perfect agreement for the other antigens (*k* = 0.81–0.93). Two criteria to determine overall HVTL-1 seropositivity improved the single antigen statistics of gag: either inclusion of env alone, or inclusion of env, tax and rex. Using a combination of gag and env for determination of overall HTLV-1 seropositivity, 100.0% specificity and 94.0% sensitivity were reached (Table 4). With the additional inclusion of tax and rex one additional serum was detected increasing the sensitivity to 95% (not shown) while maintaining specificity of 100%.

Potential cross-reactivity of HTLV-1 with HIV-1 antibody measurements was assessed by measuring antibody reactivities against HIV-1 gag, env, tat and rev (Kranz *et al*., submitted) in parallel with their homologs HTLV-1 gag, env, tax and rex. Of the 200 HTLV-1 reference sera, 10 showed detectable antibody reactivities against any of the HIV-1 antigens. As antibody patterns and responses did not correlate between HIV-1 and HTLV-1 homologs (r = -0.01 to r = 0.09), no cross-reactivity between HIV-1 and HTLV-1 could be detected based on RP III (S4 Fig).

#### *T*. *gondii* Monoplex Serology validation

*T*. *gondii* Monoplex Serology initially comprised antigens p22, sag1 and bag1, and was validated against the Sabin-Feldman dye test on reference panel IV. Both antigens p22 and sag1 showed high capacity to distinguish reference assay serostatus, with overlapping reactivities for borderline reacting sera (range: 30–110 MFI; Fig 1IV). This resulted in detection of six false-positives (12%) out of 50 reference assay negative sera for both p22 and sag1. Ten (7%) and 23 (16%) false-negatives were detected out of 148 reference assay positive sera for p22 and sag1, respectively. This resulted in a specificity of 86.0% for both p22 and sag1, and a sensitivity of 92.6% and 84.5%, respectively (Table 3). Kappa statistics of *k* = 0.77 and *k* = 0.64 for p22 and sag1 provided evidence of substantial agreement. Antigen bag1 did not show to be informative for the discrimination between reference assay negatives and positives. An AND/OR combination of *T*. *gondii* antigens p22 and sag1 to define overall *T*. *gondii* seropositivity improved specificity to 92.0% with comparable sensitivity of 91.2% (Table 4, S5 Fig). This resulted in substantial agreement with the reference assay (*k* = 0.79).

### Comparison of performance in Monoplex and Multiplex Serology

**Fig 2:**
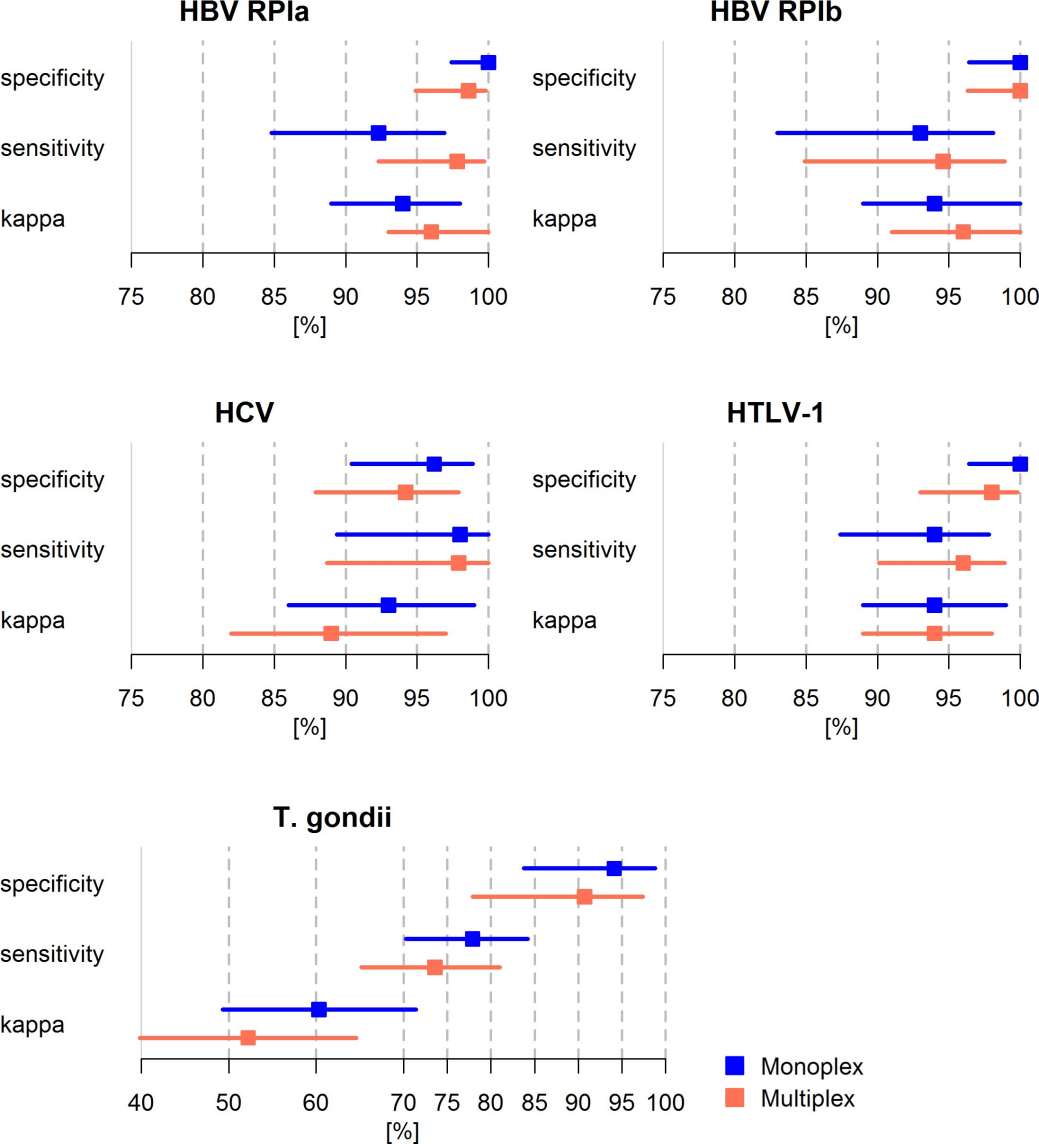
Statistical performance of pathogen-specific assays (overall seropositivity to HBV, HCV (AND/OR), HTLV-1, *T*. *gondii*) in monoplex (blue) and multiplex (orange) format. To enhance visualization, Cohen’s *kappa* statistics are illustrated as percentages. Sensitivity, specificity and Cohen’s kappa statistics are illustrated by boxes while the corresponding 95% CI is indicated by horizontal lines. Performance of Monoplex and Multiplex Serology was directly compared on the corresponding reference panel using ICCs. The comparison yielded moderate to excellent reliability between monoplex and multiplex format: ICC_HBV RPIa_: 0.94 (95% CI 0.92–0.95), ICC_HBV RPIb_: 0.97 (95% CI 0.96–0.98), ICC_HCV_: 0.97 (95% CI 0.96–0.98), ICC_HTLV-1_: 0.94 (95% CI 0.92–0.95), ICC_*T*.*gondii*_: 0.69 (95% CI 0.61–0.76). kappa: Cohen’s *kappa*. ICC: Intraclass correlation coefficient. CI: confidence interval. RP: reference panel.

To compare the assay performance between monoplex and multiplex formats, HBV, HCV, HTLV-1 and *T*. *gondii* Monoplex Serology assays were incorporated into a broad Multiplex Serology panel and re-tested at the optimum serum dilution for the majority of incorporated assays (1:1000). Assay performance in multiplex format was reassessed based on the described reference panels (Ia, Ib, II-IV). The statistical performance (specificity, sensitivity, Cohen’s *kappa)* was re-evaluated against the reference assay serostatus and compared to the corresponding pathogen-specific Monoplex Serology results (Fig 2). For some of the pathogen-specific assays, slight variations in sensitivity and specificity were detected within overlapping confidence intervals. Overall statistical performance in Multiplex Serology was maintained. Sensitivity and specificity for overall seropositivity for HBV, HCV and HTLV-1 exceeded 90% in both monoplex and multiplex assay format, maintaining high concordance with the corresponding reference assay (*k* ≥ 0.85). Sensitivity, specificity and *kappa* statistics results for *T*. *gondii* at the sub-optimal serum dilution of 1:1000 are shown and compared in Fig 2. Specificity was high (>90%) in both Monoplex and Multiplex Serology settings. However, sensitivity was impaired (<80%) also strongly reducing Cohen’s kappa in both settings (*k*_Monoplex_: 0.63; *k*_Multiplex_: 0.52).

A direct comparison of Monoplex versus Multiplex Serology performance was conducted using ICCs showing excellent reliability (ICC: 0.94–0.97) for HBV, HCV and HTLV-1. The ICC for comparison of *T*. *gondii* Monoplex and Multiplex Serology showed moderate reliability (ICC: 0.69). This was strongly influenced by the loss in sensitivity at testing dilution 1:1000 and a reduced number of samples tested in multiplex format (n = 172 as opposed to n = 198).

## Discussion

Here, we report the successful development and validation of Monoplex Serology assays for HBV, HCV, HTLV-1 and *T*. *gondii*. Overall seropositivity for each pathogen yielded a sensitivity between 91.2% and 98.0%, and a specificity between 92.0% and 100.0% in comparison to reference assays in clinical use. Each Monoplex Serology assay requires only two antigens, and can thus be efficiently incorporated into Multiplex Serology, which makes simultaneous measurement of antibody reactivities and resulting detection of seropositivities against multiple pathogens very efficient. The pathogen-specific Monoplex Serology assays were additionally combined and tested in Multiplex Serology on the respective reference serum panels including a broader infectious disease assay panel (e.g. human herpesviruses 1–8, human polyomaviruses, human papillomaviruses, human immunodeficiency virus 1). Statistical performance was generally maintained (ICC: 0.69–0.97).

We used two independent reference panels for validation of HBV Monoplex Serology; the corresponding reference assays are closely related and both measured anti-HBc. This explains the very good agreement between anti-HBc Monoplex Serology and both reference assays. In general, different combinations of anti-HBc, anti-HBs and HBsAg in blood allow distinguishing between susceptible, vaccinated, acutely or chronically HBV infected individuals, or individuals who are immune after natural infection [45, 53]. During current (i.e., acute or chronic) HBV infection or after cleared HBV infection, IgG antibodies against HBc are present. Thus, seropositivity to antigen HBc in HBV Monoplex Serology indicates an HBV infection that might be resolved, acute or chronic.

Almost perfect concordance of anti-HBe with both reference assays was observed; in addition, the anti-HBc and anti-HBe measurements in HBV Monoplex Serology showed very high correlation. In both tested reference panels, most individuals were observed to be double seropositive for HBc and HBe by HBV Monoplex Serology. This explains the high concordance of anti-HBe measurements with the reference assays. However, neither reference panel had been tested for antibodies against HBe by the reference institutions. Thus, the validity and potential meaning of anti-HBe measurements in HBV Monoplex Serology need to be evaluated in the context of known seropatterns during HBV infection. During chronic HBV infection, or in the course of clearance of acute infection, individuals develop anti-HBe antibodies [23, 53]. Thus, individuals with a cleared HBV infection are anti-HBe seropositive. In RP Ib, the majority of HBV seropositive sera (95%) were positive for anti-HBc and negative for HBsAg by the reference institution. This pattern points to a past and cleared infection which is accompanied by HBe antibodies [53]. In RP Ia, approximately one third of HBV seropositive sera was positive for anti-HBc and negative for anti-HBs marking either a phase during acute infection, or chronic infection. The remaining two thirds of HBV seropositive sera were double seropositive for anti-HBc and anti-HBs indicating past infection. In these two subgroups of HBV infected individuals, antibodies against antigen HBe may occur [53]. Thus, although not directly validated against an anti-HBe reference assay, the presence of antibodies against HBe in the two independent anti-HBc reference panels is plausible, and supports the method’s validity.

HBV vaccination is based on HBsAg alone, and thus detection of only anti-HBs antibodies marks immunity due to vaccination. Inclusion of anti-HBs measurements in HBV Monoplex Serology would not only facilitate the discrimination of the exact HBV infection status in anti-HBc positives but would also help to identify vaccinated individuals in anti-HBc seronegatives [23, 53]. Despite multiple attempts, incorporation of anti-HBs measurements in HBV Monoplex Serology was not successful. Even covalent coupling of vaccine-derived HBsAg VLPs to the beads did not enable specific detection of anti-HBs antibodies based on three independent reference serum panels. Most likely, the specific immunogenic structure of the HBsAg complexes was destroyed during the rigid covalent coupling procedure to the beads.

HCV Monoplex Serology was successfully validated against a combination of the ARCHITECT Anti-HCV assay and confirmatory ELISA Murex anti-HCV. The antigen achieving the highest concordance with the reference assay was NS3 (*k* = 0.96). All other antigens showed very high specificities, but impaired sensitivities (Fig 1II, Table 3). The ARCHITECT Anti-HCV reference assay is based on 2 antigens representing a combination of recombinant structural and non-structural HCV antigens. The recombinant antigen HCr43 is expressed in *E*. *coli* and was reported to consist of parts of NS3 and the Core antigen. The second recombinant antigen, c100-3, comprises regions of NS3 and NS4 expressed by *S*. *cervisiae* [54]. The ARCHITECT Anti-HCV assay is a chemiluminescent microparticle immunoassay (CMIA) simultaneously presenting both recombinant antigens on microparticles to the primary antibodies in the serum. Seropositivity is defined by an overall anti-HCV signal and does not differentiate between individual contributions of the recombinant antigens to the signal. Similarly, the confirmatory ELISA Murex anti-HCV uses an antigen mixture of purified HCV antigens (NS3, rAg, Core, NS4, NS5) to detect anti-HCV antibodies [55]. The principle used in both reference assays represents a considerably different approach to data analysis in HCV Monoplex Serology. High specificity of all antigens included in HCV Monoplex Serology indicates very good concordance with both reference assays for HCV serology negatives. Impaired sensitivity for all antigens except NS3 suggests two points. First, at least in this serum reference panel, antibodies against NS3 are the most frequent in HCV seropositives compared to the other HCV Monoplex Serology antigens. Secondly, the seemingly impaired sensitivity of the other HCV antigens might be indicative of true seropatterns in HCV seropositives as the reference assay does not distinguish between seropositivity against different antigens. Thus, HCV Monoplex Serology potentially provides a higher degree of detail that cannot be evaluated in comparison to this HCV reference assay.

The reference laboratory further characterised sera tested positive by the ARCHITECT Anti-HCV assay and the confirmatory ELISA (Murex anti-HCV) using the ARCHITECT HCV Ag assay (Abbott) to distinguish between past infection (ARCHITECT Anti-HCV and Murex anti-HCV double positive) and current HCV infection (triple positive: ARCHITECT Anti-HCV, Murex anti-HCV, ARCHITECT HCV Ag assay). Although HCV Monoplex Serology could not differentiate between past and current HCV infection according to the described reference assay combination (not shown), all 28 sera diagnosed with current HCV infection were correctly classified by the NS3 antigen. In addition, 26 (93%) of these sera were also seropositive against antigen Core and 21 (75%) reacted with at least two other non-structural proteins in addition to NS3. Of the 22 sera classified as past HCV infection by the reference institution, 21 (96%) were seropositive to NS3 in HCV Monoplex Serology, 17 (77%) were seropositive to antigen Core and 11 (50%) reacted with at least two other non-structural proteins in addition to NS3. These antibody patterns indicate lower seroreactivity against Core and other non-structural HCV proteins than against NS3 in individuals with past instead of current HCV infection.

Four sera were tested positive by the reference assay (ARCHITECT Anti-HCV), but negative by the confirmatory ELISA (Murex anti-HCV, Diasorin) and were thus regarded as false-positives by the reference laboratory. Of these four sera, only one reacted with antigen NS3 in HCV Monoplex Serology and none with antigen Core or at least two non-structural proteins other than NS3. Although not being representative due to low numbers, this indicates that HCV Monoplex Serology may detect less false-positives than the ARCHITECT Anti-HCV assay.

Dondog *et al*. validated HCV Monoplex Serology against the Abbott AxSYM HCV version 3.0 microparticle enzyme immune assay plus a confirmatory assay (RT-PCR or Western blot) [38]. The AxSYM assay was a precursor of the ARCHITECT Anti-HCV assay and was based on a partly overlapping set of antigens [56]. Differential performance of HCV Monoplex Serology and the resulting optimum criterion for definition of HCV seropositivity in comparison with the previous validation may be due to different characteristics of the reference assays such as the antigen panel and/or the serum panel.

HTLV-1 Monoplex Serology was successfully validated against a combination of two reference assays used for HTLV-1 diagnosis in the UK (Table 2). Single antigen performance of HTLV-1 gag and env, an AND/OR combination of gag and env, as well as a combination of all informative HTLV-1 antigens (gag, env, tax, rex) resulted in high concordance (*k*: 0.88–0.95) with the standard diagnosis algorithm in the UK. Inclusion of antigens tax and rex to determine overall HTLV-1 seropositivity classified one additional reference assay seropositive serum correctly in comparison to the gag AND/OR env combination. However, this algorithm is very detailed and likely suffers from overfitting to the underlying serum panel. Thus, it is questionable whether it is directly transferable to other studies. Therefore, for inclusion of a robust and efficient HTLV-1 assay in Multiplex Serology, antigens gag and env are considered sufficient.

Alignment of the HTLV-1 antigen rex sequence with the corresponding sequence of the NCBI database revealed 3 amino acid changes. However, no reference sequence of strain MT-2 was available for rex. Thus, these mismatches could also represent differences in the parental DNA. Statistical performance of HTLV-1 antigen rex suggests no major impairment of immunogenic epitopes.

To assess potential cross-reactivity of the included HTLV-1 antigens, the reference sera were also tested for antibodies against homologous HIV-1 antigens. However, no correlation was observed (r: -0.01 to 0.09, SF 4). Antibody patterns in HTLV-1 reference sera reacting against at least one HIV-1 antigen suggest that two HTLV-1 negative individuals are true HIV-1 positives and 8 individuals were double seropositive for HTLV-1 and HIV-1 (not shown).

*Toxoplasma gondii* Monoplex Serology was validated against the Sabin-Feldman dye test. Single antigen performance could be improved by a sag1 AND/OR p22 combination to define overall *T*. *gondii* seropositivity. The reference assay uses *T*. *gondii* tachyzoites. During this life cycle stage, five major surface antigens are expressed including p22 and sag1 [57, 58]. The 14 observed false-negatives by the sag1 AND/OR p22 criterion might be seropositive to at least one of the other three surface antigens and are thus missed by *T*. *gondii* Monoplex Serology. The observation that a considerable number of reference assay positive sera are seropositive to p22, but not against sag1 indicates that upon infection with *T*. *gondii*, the host might not elicit antibodies against all surface antigens. Thus, *T*. *gondii* Monoplex Serology could potentially be improved by including further surface antigens not expressed in this study.

*T*. *gondii* serology at both serum dilutions was shown to be highly specific (>90%). Reduced sensitivity in Monoplex and Multiplex Serology for *T*. *gondii* was observed at a 1:1000 serum dilution in comparison to 1:100. Thus, *T*. *gondii* Serology (both in monoplex and multiplex format) is recommended to be performed at serum dilution 1:100. For incorporation of *T*. *gondii* Monoplex Serology into Multiplex Serology at dilution 1:1000, it should be noted that despite missing true-positives, the detection of false-positives is expected to be very limited. Therefore, depending on the aim of the study, inclusion of *T*. *gondii* serology into Multiplex Serology at serum dilution 1:1000 might be feasible.

Our successful assay validation for HBV, HCV, HTLV-1 and *T*. *gondii* implies robustness of their use in future large-scale seroepidemiological studies. In fact, all assays have already been incorporated in Multiplex Serology [59, 60, 61].

## Supporting information

S1 Fig Correlation of anti-HBc and anti-HBe antibody measurements in Monoplex Serology based on RP Ia and Ib. r_RPIa_ = 0.98, r_RPIb_ = 0.99.
Refstat: reference status.
(JPG)

S2 Fig **Anti-HBs measurements stratified by reference serum status based on RP Va (Panels A-C) and Vb (Architect) and Vc (Centaur) (Panel D).** Except for the GST-fusion antigen (A) all antigens were obtained from external providers and covalently coupled to polystyrene beads. B: Recombinantly expressed commercial HBs antigen expressed from *Saccharomyces cerevisiae*. In panels C and D, covalently coupled HBs VLPs were tested on three reference panels (Va-c) from different reference laboratories. Reference panel Va was tested based on the same reference assay as Vb, albeit in different laboratories. Reference panels Vb and Vc were tested by different reference assays, but in the same laboratory.
VLP: virus-like particle.
S.c. Saccharomyces cerevisiae.
(JPG)

S3 Fig Graphical illustration of different criteria for overall HCV seropositivity based on antigens NS3 and Core. Red lines: optimum cut-offs for overall HCV seropositivity defined by NS3 AND/OR Core seropositivity
Green lines: optimum cut-offs for overall HCV seropositivity defined by NS3 AND Core seropositivity.
Refstat: reference status.
(JPG)

S4 Fig Correlation of measured HTLV-1 reactivities with HIV-1 reactivities based on homologous antigens gag, env, tax (tat) and rex (rev) on RP III. No correlation could be detected. r_gag_ = 0.005, r_env_ = 0.09, r_tax_ = 0.07, r_rex_ = -0.01.
(JPG)

S5 Fig Measured antibody responses against p22 and sag1 were plotted against each other and grouped by reference assay serostatus (blue triangle for positive, black box for negative). In red, optimized cut-offs are shown.
(JPG)
