# Supplementary figures and images for: Validation of Multiplex Serology for human hepatitis viruses B and C, human T-lymphotropic virus 1 and Toxoplasma gondii
Source: PLoS One. 2019 Jan 7;14(1):e0210407. doi: 10.1371/journal.pone.0210407 (PMC6322760; doi:10.1371/journal.pone.0210407)

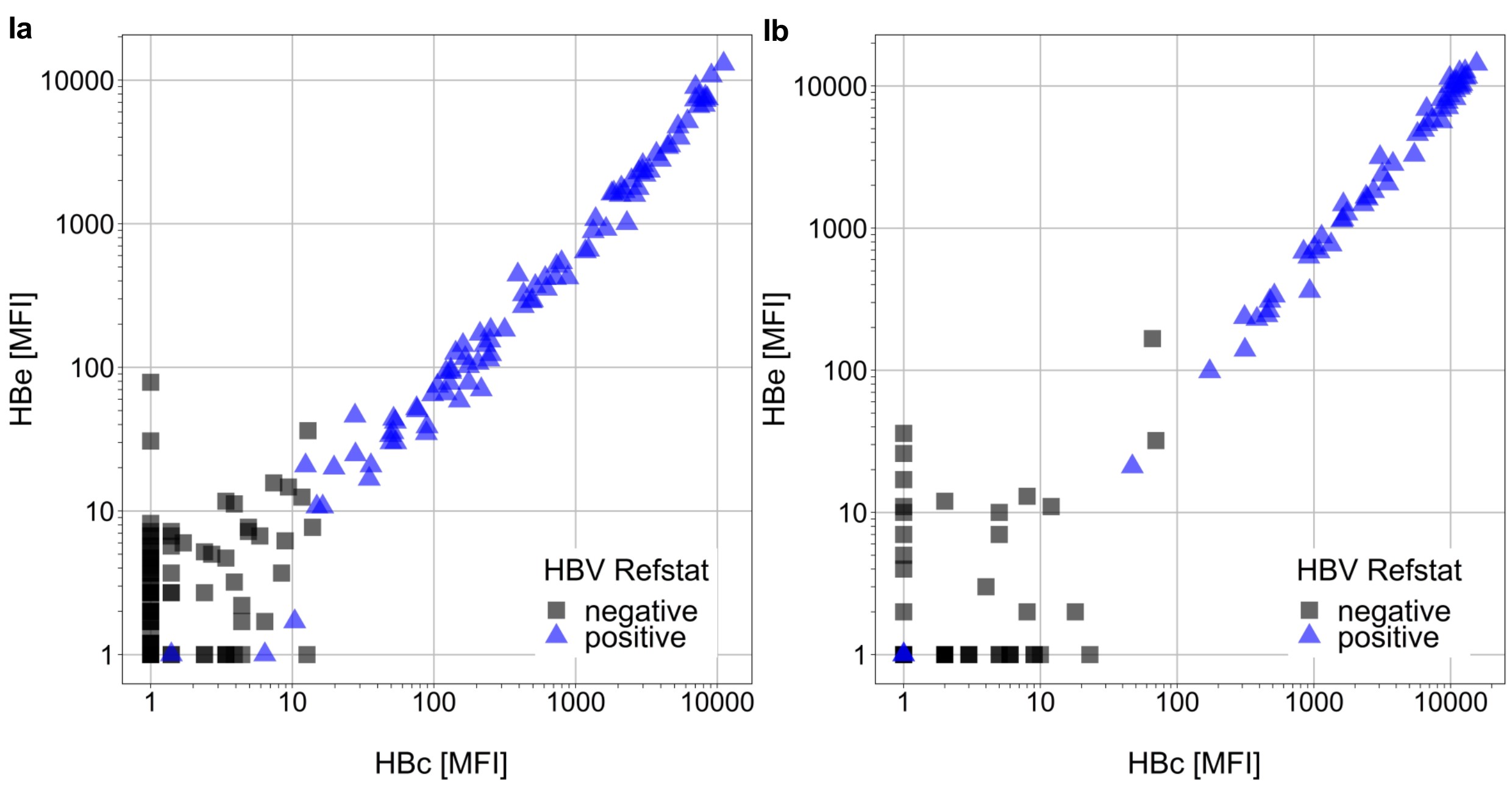

Supplement: S1 Fig — rRPIa = 0.98, rRPIb = 0.99. Refstat: reference status. (JPG) [file pone.0210407.s001.jpg]

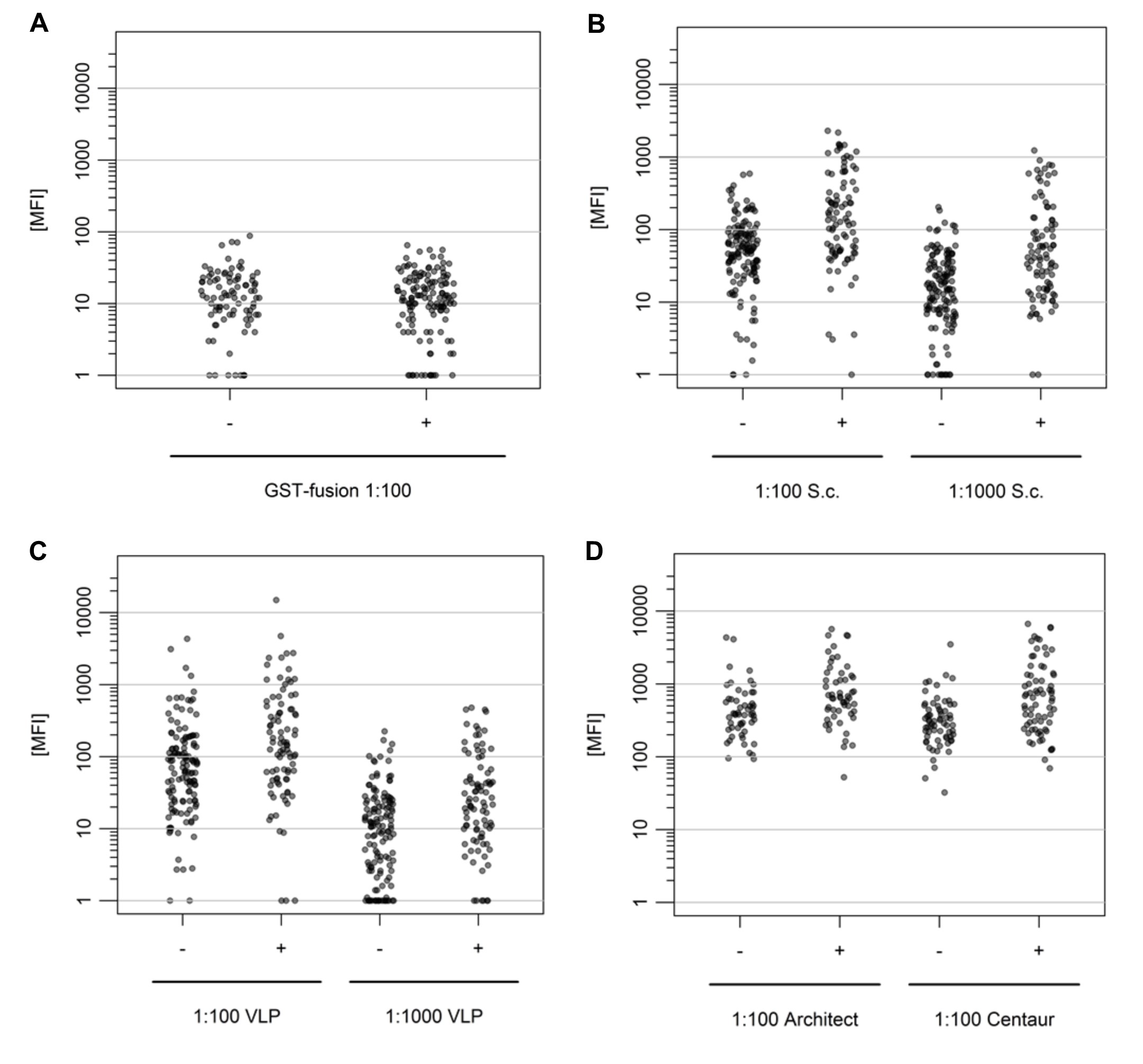

Supplement: S2 Fig — Anti-HBs measurements stratified by reference serum status based on RP Va (Panels A-C) and Vb (Architect) and Vc (Centaur) (Panel D). Except for the GST-fusion antigen (A) all antigens were obtained from external providers and covalently coupled to polystyrene beads. B: Recombinantly expressed commercial HBs antigen expressed from Saccharomyces cerevisiae. In panels C and D, covalently coupled HBs VLPs were tested on three reference panels (Va-c) from different reference laboratories. Reference panel Va was tested based on the same reference assay as Vb, albeit in different laboratories. Reference panels Vb and Vc were tested by different reference assays, but in the same laboratory. VLP: virus-like particle. S.c. Saccharomyces cerevisiae. (JPG) [file pone.0210407.s002.jpg]

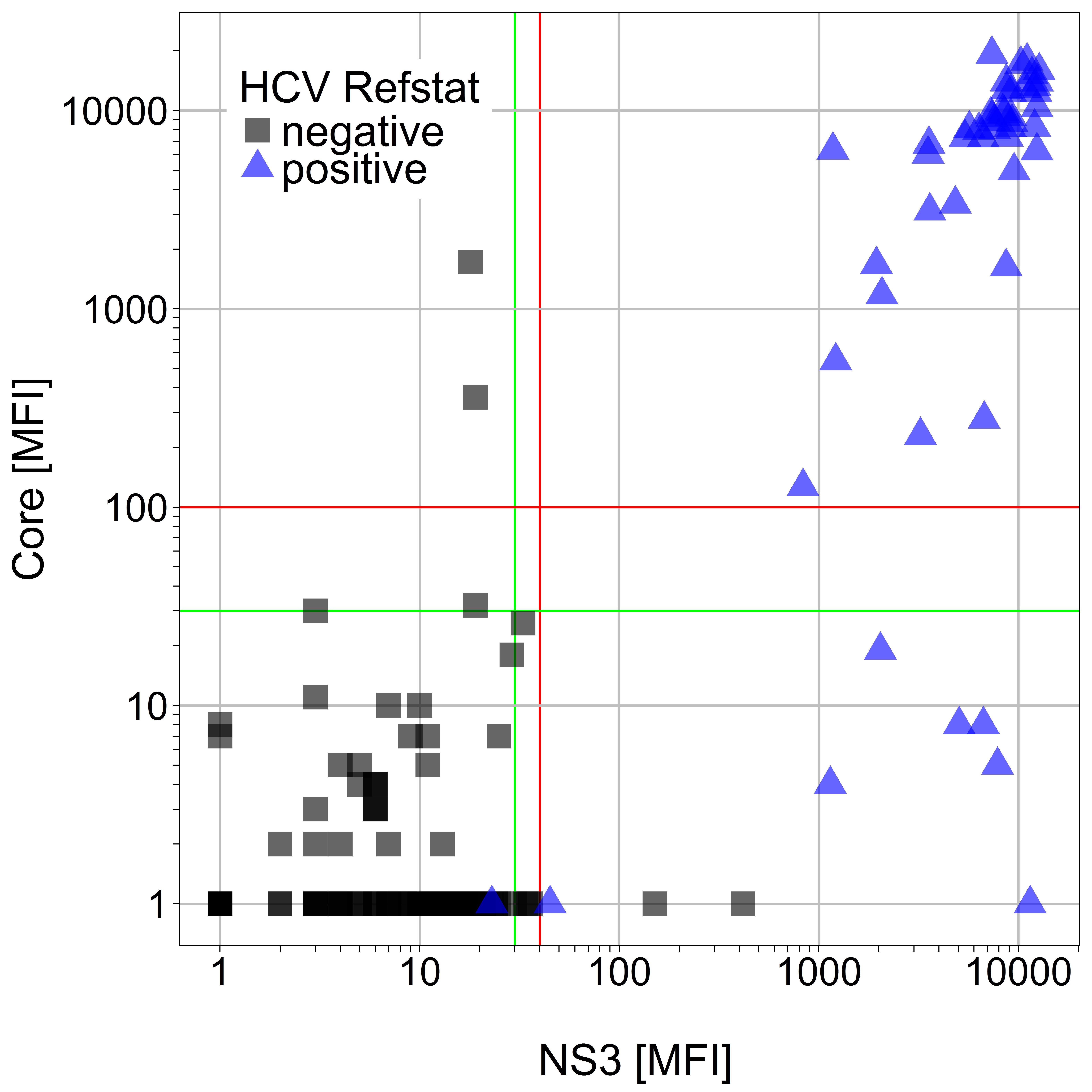

Supplement: S3 Fig — Red lines: optimum cut-offs for overall HCV seropositivity defined by NS3 AND/OR Core seropositivity Green lines: optimum cut-offs for overall HCV seropositivity defined by NS3 AND Core seropositivity. Refstat: reference status. (JPG) [file pone.0210407.s003.jpg]

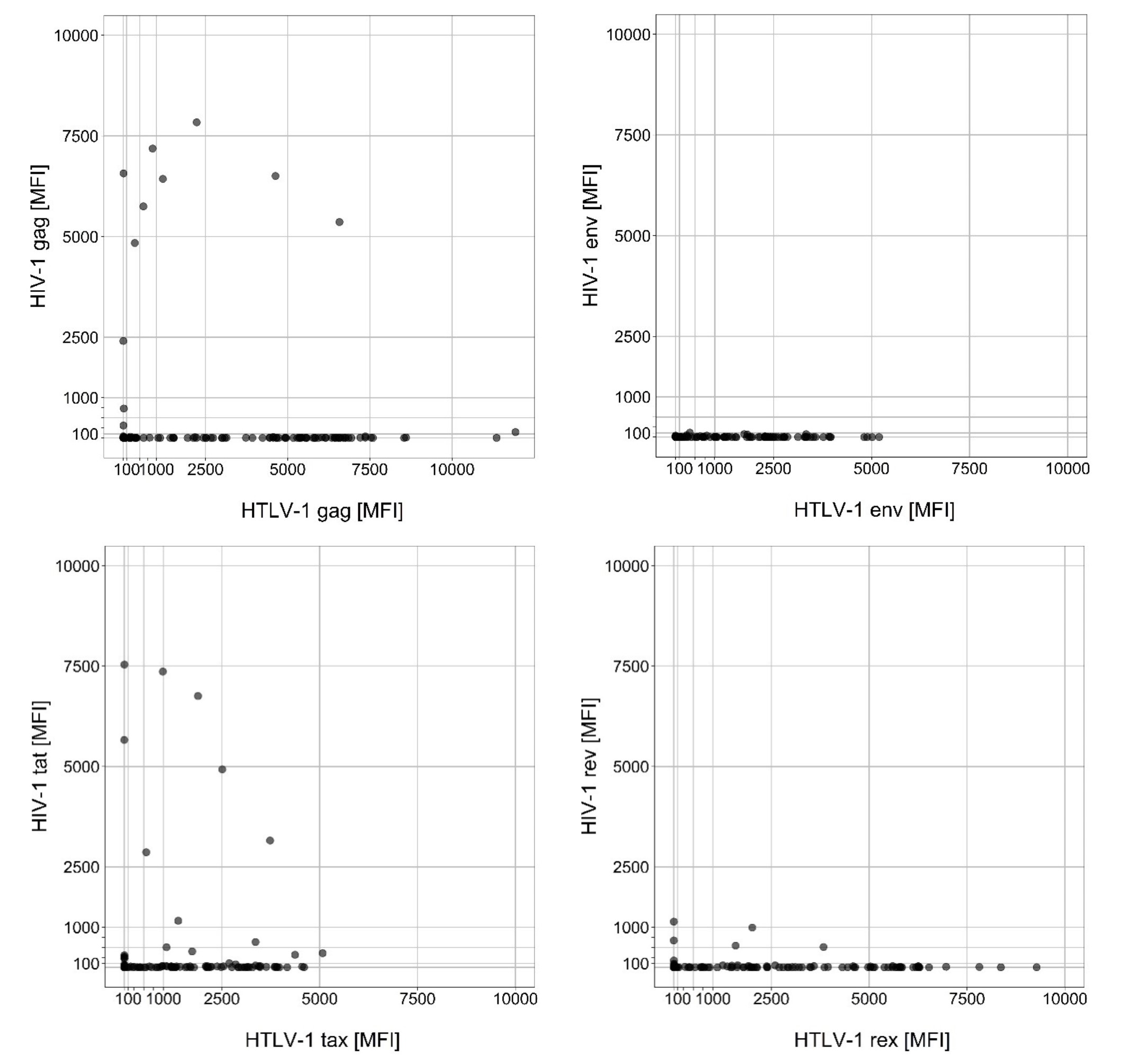

Supplement: S4 Fig — No correlation could be detected. rgag = 0.005, renv = 0.09, rtax = 0.07, rrex = -0.01. (JPG) [file pone.0210407.s004.jpg]

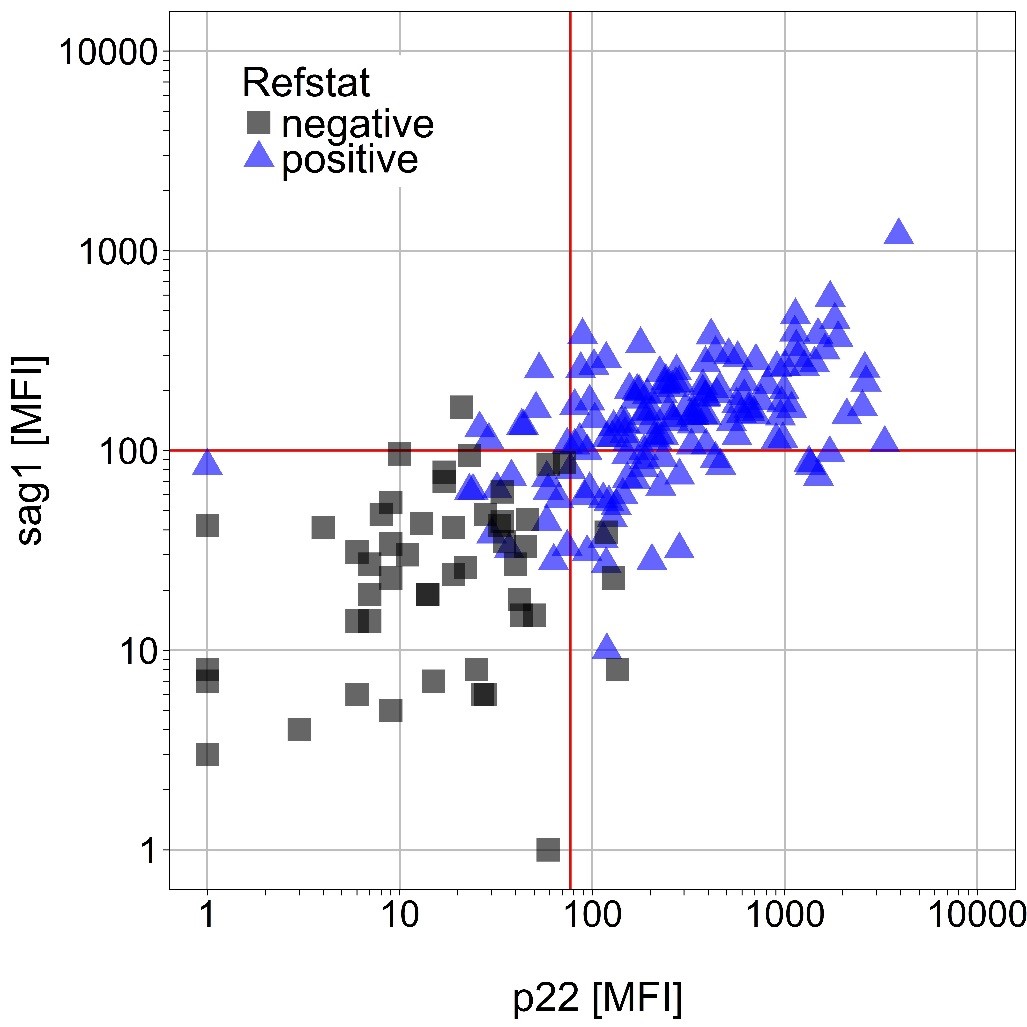

Supplement: S5 Fig — In red, optimized cut-offs are shown. (JPG) [file pone.0210407.s005.jpg]
